# Supplementary material for: Artificial intelligence as a diagnostic aid in cross-sectional radiological imaging of the abdominopelvic cavity: a protocol for a systematic review
Source: BMJ Open. 2021 Oct 19;11(10):e054411. doi: 10.1136/bmjopen-2021-054411 (PMC8529972; doi:10.1136/bmjopen-2021-054411)
Supplement: Supplementary data [file bmjopen-2021-054411supp001.pdf]

# APPENDIX S1

## Artificial intelligence as a diagnostic aid in cross-sectional imaging of the abdominopelvic cavity: A protocol for a systematic review

### Medline Search

1. artificial intelligence/ or machine learning/ or deep learning/ or supervised machine learning/ or support vector machine/ or unsupervised machine learning/
2. computer heuristics/ or expert systems/ or fuzzy logic/ or knowledge bases/ or biological ontologies/ or gene ontology/ or natural language processing/ or neural networks, computer/ or robotics/
3. Machine learning.ti,ab,kf.
4. Artificial Intelligence.ti,ab,kf.
5. Naive Bayes.ti,ab,kf.
6. bayesian learning.ti,ab,kf.
7. Neural network*.ti,ab,kf.
8. Natural language processing.ti,ab,kf.
9. support vector*.ti,ab,kf.
10. random forest*.ti,ab,kf.
11. boosting.ti,ab,kf.
12. deep learning.ti,ab,kf.
13. machine intelligence.ti,ab,kf.
14. computational intelligence.ti,ab,kf.
15. computer reasoning.ti,ab,kf.
16. 1 or 2 or 3 or 4 or 5 or 6 or 7 or 8 or 9 or 10 or 11 or 12 or 13 or 14 or 15

17. tomography/ or colonography, computed tomographic/ or computed tomography angiography/ or positron emission tomography computed tomography/
18. magnetic resonance imaging/ or cholangiopancreatography, magnetic resonance/ or diffusion magnetic resonance imaging/ or diffusion tensor imaging/ or echo-planar imaging/ or fluorine-19 magnetic resonance imaging/ or magnetic resonance angiography/ or magnetic resonance imaging, cine/ or multiparametric magnetic resonance imaging/
19. (MRI or Magnetic Resonance Imaging).ti,ab,kf.
20. 17 or 18 or 19
21. pelvis/ or lesser pelvis/ or pelvic floor/
22. (radiography adj3 abdominal).ti,ab,kf.
23. (Abdomen or abdominal cavity or peritoneum or douglas' pouch or mesentery or mesocolon or omentum or peritoneal cavity or peritoneal stomata or retroperitoneal space).ti,ab,kf.
24. (Pelvis or pelvic floor).ti,ab,kf.
25. 21 or 22 or 23 or 24
26. exp Animals/ not Humans/
27. (animal model* or rat or rats or mouse or mice or rodent* or sheep or lambs or murine or pigs or piglets or swine or porcine or rabbit or rabbits or cat or cats or feline or dog or dogs or canine or cattle or bovine or marmoset* or monkey or monkeys or trout or zebra fish*).ti.
28. (Comment or editorial or letter or case reports).pt.
29. (endoscope or gastroscope or colonoscope or capsule endoscopy or endoscopy).ti,ab,kf.
30. 26 or 27 or 28 or 29

31. 16 and 20 and 25

32. 31 not 30

33. (2012* or 2013* or 2014* or 2015* or 2016* or 2017* or 2018* or 2019* or 2020* or 202101*).yr,ed,dc,ep.

34. 32 and 33

**Embase via Ovid**

1. artificial intelligence/ or ambient intelligence/ or automated reasoning/ or computer heuristics/ or multicriteria decision analysis/
2. exp machine learning/
3. deep learning/
4. supervised machine learning/
5. exp support vector machine/
6. unsupervised machine learning/
7. hyperheuristics/ or metaheuristics/
8. expert systems/ or fuzzy logic/ or knowledge bases/ or natural language processing/ or neural networks, computer/ or robotics/
9. biological ontologies/ or gene ontology/
10. ((machine or bayesian or deep) adj1 learning).ti,ab,kw.
11. ((artificial or machine or computational) adj1 intelligence).ti,ab,kw.
12. Naive Bayes.ti,ab,kw.
13. Neural network*.ti,ab,kw.
14. Natural language processing.ti,ab,kw.
15. support vector*.ti,ab,kw.
16. random forest*.ti,ab,kw.
17. boosting.ti,ab,kw.
18. computer reasoning.ti,ab,kw.
19. 1 or 2 or 3 or 4 or 5 or 6 or 7 or 8 or 9 or 10 or 11 or 12 or 13 or 14 or 15 or 16 or 17 or 18
20. exp Tomography/

21. colonography, computed tomographic/ or computed tomography angiography/ or four-dimensional computed tomography/ or positron emission tomography computed tomography/ or single photon emission computed tomography computed tomography/ or tomography, spiral computed/

22. magnetic resonance imaging/ or cholangiopancreatography, magnetic resonance/ or diffusion magnetic resonance imaging/ or diffusion tensor imaging/ or echo-planar imaging/ or fluorine-19 magnetic resonance imaging/ or magnetic resonance angiography/ or magnetic resonance imaging, cine/ or multiparametric magnetic resonance imaging/

23. (MRI or Magnetic Resonance Imaging).ti,ab,kw.

24. 20 or 21 or 22 or 23

25. pelvis/ or lesser pelvis/ or pelvic floor/

26. (radiography adj3 abdominal).ti,ab,kw.

27. (Abdomen or abdominal cavity or peritoneum or douglas' pouch or mesentery or mesocolon or omentum or peritoneal cavity or peritoneal stomata or retroperitoneal space).ti,ab,kw.

28. (Pelvis or pelvic floor).ti,ab,kw.

29. 25 or 26 or 27 or 28

30. (Animal/ or Nonhuman/) not Human/

31. Animal Experiment/ not (Human Experiment/ or Human/)

32. (animal model* or rat or rats or mouse or mice or rodent* or sheep or lambs or murine or pigs or piglets or swine or porcine or rabbit or rabbits or cat or cats or feline or dog or dogs or canine or cattle or bovine or marmoset* or monkey or monkeys or trout or zebra fish*).ti.

33. (Comment or editorial or letter or case reports).pt.

34. (endoscope or gastroscope or colonoscope or capsule endoscopy or endoscopy).ti,ab,kw.

35. 30 or 31 or 32 or 33 or 34

36. 19 and 24 and 29

37. 36 not 35

38. (2012* or 2013* or 2014* or 2015* or 2016* or 2017* or 2018* or 2019* or 2020* or 202101*).yr,em.

39. 37 and 38

**Cochrane Central Register of Controlled Trials**

#1 MeSH descriptor: [Artificial Intelligence] this term only MeSH

#2 MeSH descriptor: [Machine Learning] this term only MeSH

#3 MeSH descriptor: [Deep Learning] this term only MeSH

#4 MeSH descriptor: [Supervised Machine Learning] this term only MeSH

#5 MeSH descriptor: [Unsupervised Machine Learning] this term only MeSH

#6 MeSH descriptor: [Support Vector Machine] this term only MeSH

#7 MeSH descriptor: [Computer Heuristics] this term only MeSH

#8 MeSH descriptor: [Expert Systems] this term only MeSH

#9 MeSH descriptor: [Fuzzy Logic] this term only MeSH

#10 MeSH descriptor: [Knowledge Bases] this term only MeSH

#11 MeSH descriptor: [Biological Ontologies] this term only MeSH

#12 MeSH descriptor: [Gene Ontology] this term only MeSH

#13 MeSH descriptor: [Natural Language Processing] this term only MeSH

#14 MeSH descriptor: [Neural Networks, Computer] this term only MeSH

#15 MeSH descriptor: [Robotics] this term only MeSH

#16 (machine learning): ti,ab,kw

#17 (artificial intelligence):ti,ab,kw

#18 (naive bayes):ti,ab,kw

#19 (bayesian learning):ti,ab,kw

#20 (neural network*):ti,ab,kw

#21 (natural language processing):ti,ab,kw

#22 (support vector*):ti,ab,kw

#23 (random forest*):ti,ab,kw

#24 (boosting):ti,ab,kw

#25 (deep learning):ti,ab,kw

#26 (machine intelligence):ti,ab,kw

#27 (computational intelligence):ti,ab,kw

#28 (computer reasoning):ti,ab,kw

#29 #1 or #2 or #3 or #4 or #5 or #6 or #7 or #8 or #9 or #10 or #11 or #12 or #13 or #14 or #15 or #16 or #17 or #18 or #19 or #20 or #21 or #22 or #23 or #24 or #25 or #26 or #27 or #28

#30 MeSH descriptor: [Tomography] this term only MeSH

#31 MeSH descriptor: [Colonography, Computed Tomographic] this term only MeSH

#32 MeSH descriptor: [Computed Tomography Angiography] this term only MeSH

#33 MeSH descriptor: [Positron-Emission Tomography] this term only MeSH

#34 MeSH descriptor: [Positron Emission Tomography Computed Tomography] this term only MeSH

#35 MeSH descriptor: [Magnetic Resonance Imaging] this term only MeSH

#36 MeSH descriptor: [Cholangiopancreatography, Magnetic Resonance] this term only MeSH

#37 MeSH descriptor: [Diffusion Magnetic Resonance Imaging] this term only MeSH

#38 MeSH descriptor: [Diffusion Tensor Imaging] this term only MeSH

#39 MeSH descriptor: [Echo-Planar Imaging] this term only MeSH

#40 MeSH descriptor: [Fluorine-19 Magnetic Resonance Imaging] this term only MeSH

#41 MeSH descriptor: [Magnetic Resonance Angiography] this term only MeSH

#42 MeSH descriptor: [Magnetic Resonance Imaging, Cine] this term only MeSH

#43 MeSH descriptor: [Multiparametric Magnetic Resonance Imaging] this term only MeSH

#44 (MRI):ti,ab,kw

#45 (magnetic resonance imaging):ti,ab,kw

#46 #30 or #31 or #32 or #33 or #34 or #35 or #36 or #37 or #38 or #39 or #40 or #41 or #42 or #43 or #44 or #45

#47 MeSH descriptor: [Pelvis] this term only MeSH

#48 MeSH descriptor: [Lesser Pelvis] this term only MeSH

#49 MeSH descriptor: [Pelvic Floor] this term only MeSH

#50 ((radiography NEAR/3 abdominal)):ti,ab,kw

#51 ((radiography NEAR/3 abdomen)):ti,ab,kw

#52 ((Abdomen or abdominal cavity or peritoneum or douglas' pouch or mesentery or mesocolon or omentum or peritoneal cavity or peritoneal stomata or retroperitoneal space)):ti,ab,kw

#53 (pelvis or pelvic floor):ti,ab,kw

#54 #47 or #48 or #49 or #50 or #51 or #52 or #53

#55 #29 and #46 and #54
