# Supplementary material for: Artificial intelligence as a diagnostic aid in cross-sectional radiological imaging of the abdominopelvic cavity: a protocol for a systematic review
Source: BMJ Open. 2021 Oct 19;11(10):e054411. doi: 10.1136/bmjopen-2021-054411 (PMC8529972; doi:10.1136/bmjopen-2021-054411)
Supplement: Author's manuscript [file bmjopen-2021-054411.draft_revisions.pdf]

BMJ Open

BMJ Open is committed to open peer review. As part of this commitment we make the peer review history of every article we publish publicly available.

When an article is published we post the peer reviewers' comments and the authors' responses online. We also post the versions of the paper that were used during peer review. These are the versions that the peer review comments apply to.

The versions of the paper that follow are the versions that were submitted during the peer review process. They are not the versions of record or the final published versions. They should not be cited or distributed as the published version of this manuscript.

BMJ Open is an open access journal and the full, final, typeset and author-corrected version of record of the manuscript is available on our site with no access controls, subscription charges or pay-per-view fees (http://bmjopen.bmj.com).

If you have any questions on BMJ Open's open peer review process please email info.bmjopen@bmj.com

BMJ Open

Artificial intelligence as a diagnostic aid in cross-sectional imaging of the abdominopelvic cavity: A protocol for a systematic review

Journal:	*BMJ Open*
Manuscript ID	bmjopen-2021-054411
Article Type:	Protocol
Date Submitted by the Author:	10-Jun-2021

Complete List of Authors:	Fowler, George; University of Bristol, Centre for Surgical Research, Population Health Sciences, Bristol Medical School<br>Macefield, Rhiannon; University of Bristol, Centre for Surgical Research, Population Health Sciences, Bristol Medical School<br>Hardacre, Conor; University of Bristol, Centre for Surgical Research, Population Health Sciences, Bristol Medical School<br>Callaway, Mark; Bristol Royal Infirmary, Department of Clinical Radiology<br>Smart , Neil ; Royal Devon and Exeter NHS Foundation Trust, Exeter Surgical Health Services Research Unit (HeSRU)<br>Blencowe, Natalie; University of Bristol, Centre for Surgical Research, Population Health Sciences, Bristol Medical School

Keywords:	Diagnostic radiology < RADIOLOGY & IMAGING, SURGERY, Computed tomography < RADIOLOGY & IMAGING, Magnetic resonance imaging < RADIOLOGY & IMAGING

SCHOLARONE™
Manuscripts

I, the Submitting Author has the right to grant and does grant on behalf of all authors of the Work (as defined in the below author licence), an exclusive licence and/or a non-exclusive licence for contributions from authors who are: i) UK Crown employees; ii) where BMJ has agreed a CC-BY licence shall apply, and/or iii) in accordance with the terms applicable for US Federal Government officers or employees acting as part of their official duties; on a worldwide, perpetual, irrevocable, royalty-free basis to BMJ Publishing Group Ltd ("BMJ") its licensees and where the relevant Journal is co-owned by BMJ to the co-owners of the Journal, to publish the Work in this journal and any other BMJ products and to exploit all rights, as set out in our licence.

The Submitting Author accepts and understands that any supply made under these terms is made by BMJ to the Submitting Author unless you are acting as an employee on behalf of your employer or a postgraduate student of an affiliated institution which is paying any applicable article publishing charge ("APC") for Open Access articles. Where the Submitting Author wishes to make the Work available on an Open Access basis (and intends to pay the relevant APC), the terms of reuse of such Open Access shall be governed by a Creative Commons licence – details of these licences and which Creative Commons licence will apply to this Work are set out in our licence referred to above.

Other than as permitted in any relevant BMJ Author's Self Archiving Policies, I confirm this Work has not been accepted for publication elsewhere, is not being considered for publication elsewhere and does not duplicate material already published. I confirm all authors consent to publication of this Work and authorise the granting of this licence.

For peer review only

Artificial intelligence as a diagnostic aid in cross-sectional imaging of the abdominopelvic cavity: A protocol for a systematic review

Authors: George E Fowler[1]

George.fowler@bristol.ac.uk

Rhiannon C Macefield[1]

r.macefield@bristol.ac.uk

Conor Hardacre[1]

ch17008@bristol.ac.uk

Mark P Callaway[2]

Mark.Callaway@uhbw.nhs.uk

Neil J Smart[3]

n.smart@nhs.net

Natalie S Blencowe[1]

natalie.blencowe@bristol.ac.uk

Corresponding author: George E Fowler

University of Bristol, Centre for Surgical Research, Population Health Sciences, Bristol Medical School.

For peer review only

Institutions: [1]University of Bristol, Centre for Surgical Research, Population Health Sciences, Bristol Medical School.

[2]Department of Clinical Radiology, Bristol Royal Infirmary, Bristol, UK.

[3]Exeter Surgical Health Services Research Unit (HeSRU), Royal Devon and Exeter Hospital, Exeter, UK.

ORCID ID(s): GEF (0000-0002-4133-802X)

RCM (0000-0002-6606-5427)

CH (0000-0001-9800-0479)

MPC

NJS (0000-0002-3043-8324)

NSB (0000-0002-6111-2175)

Author contributions: GEF and NSB conceived the idea for this systematic review. All authors developed the systematic review protocol. GEF drafted the manuscript (guarantor of review) and all authors critically revised the manuscript and approved the final version.

Acknowledgements: The authors would like to thank Catherine Borwick, our professional librarian, for her expertise in developing the search strategy. The authors are also grateful to Dr

For peer review only

Christin Hoffmann for leading patient and public feedback sessions as part of the wider programme of this research.

Funding statement: This work was supported by the Medical Research Council (MR/S001751/1).

Conflict of Interest: The authors have no conflicts of interest to declare.

Word count: 1,355 words

For peer review only

ABSTRACT

Introduction: The application of artificial intelligence (AI) technologies as a diagnostic aid in healthcare is increasing. Benefits include applications to improve health systems, such as rapid and accurate interpretation of medical images. This may improve the performance of diagnostic, prognostic and management decisions. While a large amount of work has been undertaken discussing the role of AI little is understood regarding the performance of such applications in the clinical setting. This systematic review aims to critically appraise the diagnostic performance of AI algorithms to identify disease from cross-sectional radiological images of the abdominopelvic cavity, to identify current limitations and inform future research.

Methods and analysis: A systematic search will be conducted on Medline, EMBASE and the Cochrane Central Register of Controlled Trials (CENTRAL) to identify relevant studies. Primary studies where AI-based technologies have been used as a diagnostic aid in cross-sectional radiological images of the abdominopelvic cavity will be included. Diagnostic accuracy of AI models, including reported sensitivity, specificity, predictive values, likelihood ratios and the area under the receiver operating characteristic curve will be examined and compared to standard practice. Risk of bias of included studies will be assessed using the QUADAS-2 tool. Findings will be reported according to the Synthesis Without Meta-analysis (SWiM) guidelines.

Ethics and dissemination: No ethical approval is required as primary data will not be collected. The results will inform further research studies in this field. Findings will be

For peer review only

disseminated at relevant conferences, on social media and published in a peer-reviewed journal.

PROSPERO registration number: CRD42021237249.

Key words: Systematic review, artificial intelligence, diagnostic accuracy, radiological imaging, abdominopelvic cavity.

For peer review only

BOX: Strengths and limitations of this study

- This will be the first systematic review to evaluate the diagnostic performance of AI models using cross-sectional radiological images of the abdominopelvic cavity, identifying current limitations and evidence gaps, and thereby focusing future research efforts.
- Robust methodology will be undertaken including duplicate screening, data extraction and of risk of bias assessment.
- Findings may be limited by the inclusion of English language publications only.

For peer review only

INTRODUCTION

In an era of ‘Big Data’, rapid developments in artificial intelligence (AI)-based technologies in medicine offer great potential to transform healthcare and improve patient outcomes [1]. The widespread adoption of digital data in healthcare has provided a vast amount of data to enable computer algorithms to extract relevant information and recognise complex patterns [2]. This includes quantitative (e.g. laboratory values) and qualitative (e.g. text-based electronic health records) data, as well as audio-visual data obtained from recordings from medical devices (e.g. electrocardiograms, digital dictation, etc). In a recent review, AI technologies were summarised as having an impact at three levels; clinicians, health systems and patients [3]. For clinicians, AI technologies can help interpret images more rapidly and accurately improving the performance of diagnostic, prognostic and management decisions [4]. For health systems, AI applications can improve workflow (e.g. administrative jobs such as scheduling of operating rooms and clinic appointments). For patients, AI technologies can provide an opportunity for individuals to process their own data to promote health (e.g. a smartwatch algorithm to detect a heart arrhythmia and the patient seeking appropriate medical attention) [3].

Medical imaging is considered a valuable source of diagnostic, prognostic and surveillance information. It also provides a pivotal role in supporting clinicians to perform procedural tasks. Images, however, have traditionally been dependent on human interpretation and there is an increasingly limited number of interpreters [5]. There has been a surge of research exploring how AI technologies can be applied to medical images to support clinicians and provide greater efficacy and efficiency in

For peer review only

clinical care [6]. One of the most promising clinical applications of AI has been in diagnostic imaging [7], particularly for radiological [7–14] and endoscopic [15,16] investigations. AI diagnostic models have been used to detect pulmonary nodules [12], liver lesions [8], pancreatic cancer [9], colorectal cancer [15] and hip fractures [13,14]. These advancements can be stratified by imaging modality (e.g., ultrasound, radiography, computed tomography (CT), magnetic resonance imaging (MRI)) and anatomical region (e.g. head and neck, thorax, abdomen and pelvis, upper and lower limbs) [17], organ [7] or speciality. However, most AI studies are currently a proof-of-concept, rather than a model deployed in the clinical setting to explore the potential benefit. Few prospective studies and RCTs evaluating the application of AI have been undertaken, and those which exist are at high risk of bias [18].

Evidence from some clinical specialities, including neurosurgery [19] and gastroenterology [20] offer insight into some of the promises and pitfalls for AI technologies in healthcare. Pitfalls include AI algorithms that can be difficult or impossible to interpret (referred to as “black box” techniques) and requiring large amounts of high quality data which can be difficult to access, especially across institutions [19]. A review of the diagnostic accuracy of AI in radiological imagining of the abdominopelvic cavity is lacking. This could benefit a variety of different surgical specialities which employ diagnostic imaging for the abdominopelvic cavity. This systematic review aims to summarise the current research and critically appraise the diagnostic performance of AI models to diagnose disease from cross-sectional radiological images of the abdominopelvic cavity which may warrant an ‘invasive procedure’ [21] for ‘therapeutic intent’. This will be compared to standard practice. The

For peer review only

quality of this research will also be assessed, to identify current limitations and inform future research efforts.

For peer review only

METHODS

Protocol and registration

This protocol has been developed in accordance with the Preferred Reporting Items for Systematic Reviews and Meta-analysis Protocols (PRISMA-P) guidelines [22]. The systematic review has been registered with the International Prospective Register of Systematic Reviews (PROSPERO; CRD42021237249).

For peer review only

Eligibility criteria

Primary research studies will be considered for eligibility using the PIRT framework (participants, index test(s), reference standard and target condition) [23]:

Participants

Adults with abdominopelvic cavity pathology diagnosed from cross-sectional radiological imaging confined to CT, MRI and positron emission tomography (PET). Studies reporting endoscopy as an imaging modality will not be included in this review, as several reviews have already explored the performance of AI in this area [16, 26].

Index test

Studies considering AI models as an intervention with the aim to provide a diagnosis.

Reference standard

Standard practice.

Target condition

Abdominopelvic cavity pathology which has had, or may warrant, an 'invasive procedure' [21] for 'therapeutic intent'.

Exclusion criteria for the studies as follows:

1. Secondary research studies (e.g. editorials and systematic reviews), case reports and case series.
2. Absence of full text publications (e.g. conference abstracts).
3. Non-English articles.
4. Animal studies.

Outcome

The primary outcome is to evaluate the diagnostic performance of AI models using cross-sectional radiological images of the abdominopelvic cavity. The diagnostic performance will be referred to as previously defined: "the ability of a test to discriminate between the target condition and health" [24]. Diagnostic measures of accuracy will include reported sensitivity, specificity and the area under the receiver operating characteristic curve (AUC).

Information sources

An electronic search of OVID SP versions of Medline, EMBASE and the Cochrane Central Register of Controlled Trials (CENTRAL) will identify all potentially relevant studies published since January 1st, 2012, using a pre-defined search strategy (Appendix S1). The cut-off from January 1st, 2012, is to accommodate for the advancement in machine learning performance with the development of deep learning approaches, an approach previously adopted in the literature [5].

For peer review only

Search strategy and study selection

The search syntax will be developed with guidance from a professional librarian using free text and Medical Subject Headings (MeSH) related to “artificial intelligence”, “diagnostic imaging” and the “abdominopelvic cavity”. Database search results will be imported into the EndNote reference management software and duplicates will be removed.

Assessment of study eligibility will be a two-stage process. First, titles and abstracts will be screened for inclusion by two independent reviewers. Any identified conflicts will be resolved through discussion, including with the wider study team if required. Final eligibility will be assessed by full-text review of potentially eligible studies by the same process. The screening will be facilitated by Rayyan software [25].

Data extraction and management

Eligible studies will undergo data extraction using a pre-designed standardized proforma and data management software (REDCap version 9.5.23). This will be done independently by at least two reviewers for at least 10% of the articles and then verified by a second thereafter. A standardized form will be used, which will include the following categories:

1. Study characteristics: first author, journal, year of publication, country of origin, study design (e.g. case control, randomised control trials etc) reporting of ethical approval, regulatory approval (e.g. Medicines and Healthcare products Regulatory Agency) and patient and public involvement.

For peer review only

2. Patient characteristics: pathology studied and surgical speciality of pathology.
3. Input features: modality of radiological imaging (e.g. CT, MRI and PET), AI model used, size of training model, comparator group used and size of this data set.
4. Outcomes: diagnostic measures of accuracy and method of validation.

Risk of bias

Risk of bias of the primary diagnostic accuracy studies will be assessed using the QUADAS-2 tool [26]. This will be done independently by at least two authors of the study and disagreements resolved by the study team.

Data Synthesis

Search results and study selection will be presented in accordance with the Preferred Reporting Items for Systematic Reviews (PRISMA) guidelines [27]. Due to the broad nature of the PIRT and anticipated high levels of heterogeneity for the primary outcome a meta-analysis of data is not planned. Findings will be presented as a descriptive summary and narrative synthesis and will be reported according to the Synthesis Without Meta-analysis guidelines [28]. The narrative synthesis will focus on the primary outcome with studies grouped by the modality of cross-sectional radiological imaging, pathology studied and surgical subspecialty.

Patient and public involvement

As part of the wider programme of work (Bristol Biomedical Research Centre, NIHR Bristol BRC), patients and the public were consulted on their views of AI being used

For peer review only

to guide doctors to make decisions about treatment. Patient and public involvement (PPI) will be sought for the dissemination of this systematic review.

Ethics and dissemination

Ethical approval is not required for the systematic review, as no primary data is collected. The review will be disseminated at relevant conferences, on social media and published in a peer-review journal.

For peer review only

References

1 He J, Baxter SL, Xu J, *et al.* The practical implementation of artificial intelligence technologies in medicine. *Nat Med* 2019;**25**:30–6. doi:10.1038/s41591-018-0307-0

2 Alloghani M, Al-Jumeily D, Aljaaf AJ, *et al.* The Application of Artificial Intelligence Technology in Healthcare: A Systematic Review. *Commun Comput Inf Sci* 2020;**1174 CCIS**:248–61. doi:10.1007/978-3-030-38752-5_20

3 Topol EJ. High-performance medicine: the convergence of human and artificial intelligence. *Nat Med* 2019;**25**:44–56. doi:10.1038/s41591-018-0300-7

4 Ben-Israel D, Jacobs WB, Casha S, *et al.* The impact of machine learning on patient care: A systematic review. *Artif Intell Med* 2020;**103**:101785. doi:10.1016/j.artmed.2019.101785

5 Liu X, Faes L, Kale AU, *et al.* A comparison of deep learning performance against health-care professionals in detecting diseases from medical imaging: a systematic review and meta-analysis. *Lancet Digit Heal* 2019;**1**:e271–97. doi:10.1016/S2589-7500(19)30123-2

6 Hosny A, Parmar C, Quackenbush J, *et al.* Artificial intelligence in radiology. *Nat Rev Cancer* 2018;**18**:500–10. doi:10.1038/s41568-018-0016-5.Artificial

7 Lundervold AS, Lundervold A. An overview of deep learning in medical imaging focusing on MRI. *Z Med Phys* 2019;**29**:102–27. doi:10.1016/j.zemedi.2018.11.002

8 Azer SA. Deep learning with convolutional neural networks for identification of liver masses and hepatocellular carcinoma: A systematic review. *World J Gastrointest Oncol* 2019;**11**:1218–30. doi:10.4251/wjgo.v11.i12.1218

9 Liu KL, Wu T, Chen PT, *et al.* Deep learning to distinguish pancreatic cancer

For peer review only

tissue from non-cancerous pancreatic tissue: a retrospective study with cross-racial external validation. *Lancet Digit Heal* 2020;**2**:e303–13. doi:10.1016/S2589-7500(20)30078-9

10 Lakshmanaprabu SK, Mohanty SN, Shankar K, *et al.* Optimal deep learning model for classification of lung cancer on CT images. *Futur Gener Comput Syst* 2019;**92**:374–82. doi:10.1016/j.future.2018.10.009

11 Titano JJ, Badgeley M, Schefflein J, *et al.* Automated deep-neural-network surveillance of cranial images for acute neurologic events. *Nat Med* 2018;**24**:1337–41. doi:10.1038/s41591-018-0147-y

12 Nam JG, Park S, Hwang EJ, *et al.* Development and validation of deep learning-based automatic detection algorithm for malignant pulmonary nodules on chest radiographs. *Radiology* 2019;**290**:218–28. doi:10.1148/radiol.2018180237

13 Gale W, Oakden-Rayner L, Carneiro G, *et al.* Detecting hip fractures with radiologist-level performance using deep neural networks. Published Online First: 2017.http://arxiv.org/abs/1711.06504

14 Lindsey R, Daluiski A, Chopra S, *et al.* Deep neural network improves fracture detection by clinicians. *Proc Natl Acad Sci U S A* 2018;**115**:11591–6. doi:10.1073/pnas.1806905115

15 Zhou D, Tian F, Tian X, *et al.* Diagnostic evaluation of a deep learning model for optical diagnosis of colorectal cancer. *Nat Commun* 2020;**11**:1–9. doi:10.1038/s41467-020-16777-6

16 Hassan C, Spadaccini M, Iannone A, *et al.* Performance of artificial intelligence for colonoscopy regarding adenoma and polyp detection: a meta-analysis. *Gastrointest Endosc* Published Online First: 2020.

For peer review only

doi:10.1016/j.gie.2020.06.059

17 Pesapane F, Codari M, Sardanelli F. Artificial intelligence in medical imaging: threat or opportunity? Radiologists again at the forefront of innovation in medicine. *Eur Radiol Exp* 2018;**2**. doi:10.1186/s41747-018-0061-6

18 Raffort J, Adam C, Carrier M, *et al.* Artificial intelligence in abdominal aortic aneurysm. *J Vasc Surg* 2020;**72**:321-333.e1. doi:10.1016/j.jvs.2019.12.026

19 Senders JT, Arnaout O, Karhade A V., *et al.* Natural and artificial intelligence in neurosurgery: A systematic review. *Clin Neurosurg* 2018;**83**:181–92. doi:10.1093/neuros/nyx384

20 Yang YJ, Bang CS. Application of artificial intelligence in gastroenterology. *World J Gastroenterol* 2019;**25**:1666–83. doi:10.3748/wjg.v25.i14.1666

21 Cousins S, Blencowe NS, Blazeby JM. What is an invasive procedure? A definition to inform study design, evidence synthesis and research tracking. *BMJ Open* 2019;**9**:2018–20. doi:10.1136/bmjopen-2018-028576

22 Moher D, Shamseer L, Clarke M, *et al.* Preferred reporting items for systematic review and meta-analysis protocols (PRISMA-P) 2015 statement. *Syst Rev* 2015;**4**:1–9.

23 Jj, Deeks, S, Wisniewski DC, Diagnostic C, Accuracy T, *et al.* Guide to the contents of a Cochrane Diagnostic Test Accuracy Protocol. 2013;:1–15.

24 Simundic AM. Measures of Diagnostic Accuracy: Basic Definitions. *EJIFCC* 2009;**19**:203–11.https://www.ncbi.nlm.nih.gov/pubmed/27683318%0Ahttps://www.ncbi.nlm.nih.gov/pmc/articles/PMC4975285/pdf/ejifcc-19-203.pdf

25 Ouzzani M, Hammady H, Fedorowicz Z, *et al.* Rayyan-a web and mobile app for systematic reviews. *Syst Rev* 2016;**5**:1–10. doi:10.1186/s13643-016-0384-

For peer review only

4

26 Whiting PF, Weswood ME, Rutjes AWS, *et al.* Evaluation of QUADAS, a tool for the quality assessment of diagnostic accuracy studies. *BMC Med Res Methodol* 2006;**6**. doi:10.1186/1471-2288-6-9

27 McInnes MDF, Moher D, Thombs BD, *et al.* Preferred Reporting Items for a Systematic Review and Meta-analysis of Diagnostic Test Accuracy Studies The PRISMA-DTA Statement. *JAMA - J Am Med Assoc* 2018;**319**:388–96. doi:10.1001/jama.2017.19163

28 Campbell M, McKenzie JE, Sowden A, *et al.* Synthesis without meta-analysis (SWiM) in systematic reviews: Reporting guideline. *BMJ* 2020;**368**:1–6. doi:10.1136/bmj.l6890

For peer review only

APPENDIX S1

Artificial intelligence as a diagnostic aid in cross-sectional imaging of the abdominopelvic cavity: A protocol for a systematic review

Medline Search

1. artificial intelligence/ or machine learning/ or deep learning/ or supervised machine learning/ or support vector machine/ or unsupervised machine learning/

2. computer heuristics/ or expert systems/ or fuzzy logic/ or knowledge bases/ or biological ontologies/ or gene ontology/ or natural language processing/ or neural networks, computer/ or robotics/

3. Machine learning.ti,ab,kf.

4. Artificial Intelligence.ti,ab,kf.

5. Naive Bayes.ti,ab,kf.

6. bayesian learning.ti,ab,kf.

7. Neural network*.ti,ab,kf.

8. Natural language processing.ti,ab,kf.

9. support vector*.ti,ab,kf.

10. random forest*.ti,ab,kf.

11. boosting.ti,ab,kf.

12. deep learning.ti,ab,kf.

13. machine intelligence.ti,ab,kf.

14. computational intelligence.ti,ab,kf.

15. computer reasoning.ti,ab,kf.

16. 1 or 2 or 3 or 4 or 5 or 6 or 7 or 8 or 9 or 10 or 11 or 12 or 13 or 14 or 15

For peer review only

17. tomography/ or colonography, computed tomographic/ or computed tomography angiography/ or positron emission tomography computed tomography/

18. magnetic resonance imaging/ or cholangiopancreatography, magnetic resonance/ or diffusion magnetic resonance imaging/ or diffusion tensor imaging/ or echo-planar imaging/ or fluorine-19 magnetic resonance imaging/ or magnetic resonance angiography/ or magnetic resonance imaging, cine/ or multiparametric magnetic resonance imaging/

19. (MRI or Magnetic Resonance Imaging).ti,ab,kf.

20. 17 or 18 or 19

21. pelvis/ or lesser pelvis/ or pelvic floor/

22. (radiography adj3 abdominal).ti,ab,kf.

23. (Abdomen or abdominal cavity or peritoneum or douglas' pouch or mesentery or mesocolon or omentum or peritoneal cavity or peritoneal stomata or retroperitoneal space).ti,ab,kf.

24. (Pelvis or pelvic floor).ti,ab,kf.

25. 21 or 22 or 23 or 24

26. exp Animals/ not Humans/

27. (animal model* or rat or rats or mouse or mice or rodent* or sheep or lambs or murine or pigs or piglets or swine or porcine or rabbit or rabbits or cat or cats or feline or dog or dogs or canine or cattle or bovine or marmoset* or monkey or monkeys or trout or zebra fish*).ti.

28. (Comment or editorial or letter or case reports).pt.

29. (endoscope or gastroscope or colonoscope or capsule endoscopy or endoscopy).ti,ab,kf.

30. 26 or 27 or 28 or 29

For peer review only

31. 16 and 20 and 25

32. 31 not 30

33. (2012* or 2013* or 2014* or 2015* or 2016* or 2017* or 2018* or 2019* or 2020* or 202101*).yr,ed,dc,ep.

34. 32 and 33

For peer review only

Embase via Ovid

1. artificial intelligence/ or ambient intelligence/ or automated reasoning/ or computer heuristics/ or multicriteria decision analysis/

2. exp machine learning/

3. deep learning/

4. supervised machine learning/

5. exp support vector machine/

6. unsupervised machine learning/

7. hyperheuristics/ or metaheuristics/

8. expert systems/ or fuzzy logic/ or knowledge bases/ or natural language processing/ or neural networks, computer/ or robotics/

9. biological ontologies/ or gene ontology/

10. ((machine or bayesian or deep) adj1 learning).ti,ab,kw.

11. ((artificial or machine or computational) adj1 intelligence).ti,ab,kw.

12. Naive Bayes.ti,ab,kw.

13. Neural network*.ti,ab,kw.

14. Natural language processing.ti,ab,kw.

15. support vector*.ti,ab,kw.

16. random forest*.ti,ab,kw.

17. boosting.ti,ab,kw.

18. computer reasoning.ti,ab,kw.

19. 1 or 2 or 3 or 4 or 5 or 6 or 7 or 8 or 9 or 10 or 11 or 12 or 13 or 14 or 15 or 16 or 17 or 18

20. exp Tomography/

For peer review only

21. colonography, computed tomographic/ or computed tomography angiography/ or four-dimensional computed tomography/ or positron emission tomography computed tomography/ or single photon emission computed tomography computed tomography/ or tomography, spiral computed/

22. magnetic resonance imaging/ or cholangiopancreatography, magnetic resonance/ or diffusion magnetic resonance imaging/ or diffusion tensor imaging/ or echo-planar imaging/ or fluorine-19 magnetic resonance imaging/ or magnetic resonance angiography/ or magnetic resonance imaging, cine/ or multiparametric magnetic resonance imaging/

23. (MRI or Magnetic Resonance Imaging).ti,ab,kw.

24. 20 or 21 or 22 or 23

25. pelvis/ or lesser pelvis/ or pelvic floor/

26. (radiography adj3 abdominal).ti,ab,kw.

27. (Abdomen or abdominal cavity or peritoneum or douglas' pouch or mesentery or mesocolon or omentum or peritoneal cavity or peritoneal stomata or retroperitoneal space).ti,ab,kw.

28. (Pelvis or pelvic floor).ti,ab,kw.

29. 25 or 26 or 27 or 28

30. (Animal/ or Nonhuman/) not Human/

31. Animal Experiment/ not (Human Experiment/ or Human/)

32. (animal model* or rat or rats or mouse or mice or rodent* or sheep or lambs or murine or pigs or piglets or swine or porcine or rabbit or rabbits or cat or cats or feline or dog or dogs or canine or cattle or bovine or marmoset* or monkey or monkeys or trout or zebra fish*).ti.

33. (Comment or editorial or letter or case reports).pt.

For peer review only

34. (endoscope or gastroscope or colonoscope or capsule endoscopy or endoscopy).ti,ab,kw.

35. 30 or 31 or 32 or 33 or 34

36. 19 and 24 and 29

37. 36 not 35

38. (2012* or 2013* or 2014* or 2015* or 2016* or 2017* or 2018* or 2019* or 2020* or 202101*).yr,em.

39. 37 and 38

For peer review only

Cochrane Central Register of Controlled Trials

#1 MeSH descriptor: [Artificial Intelligence] this term only MeSH

#2 MeSH descriptor: [Machine Learning] this term only MeSH

#3 MeSH descriptor: [Deep Learning] this term only MeSH

#4 MeSH descriptor: [Supervised Machine Learning] this term only MeSH

#5 MeSH descriptor: [Unsupervised Machine Learning] this term only MeSH

#6 MeSH descriptor: [Support Vector Machine] this term only MeSH

#7 MeSH descriptor: [Computer Heuristics] this term only MeSH

#8 MeSH descriptor: [Expert Systems] this term only MeSH

#9 MeSH descriptor: [Fuzzy Logic] this term only MeSH

#10 MeSH descriptor: [Knowledge Bases] this term only MeSH

#11 MeSH descriptor: [Biological Ontologies] this term only MeSH

#12 MeSH descriptor: [Gene Ontology] this term only MeSH

#13 MeSH descriptor: [Natural Language Processing] this term only MeSH

#14 MeSH descriptor: [Neural Networks, Computer] this term only MeSH

#15 MeSH descriptor: [Robotics] this term only MeSH

#16 (machine learning): ti,ab,kw

#17 (artificial intelligence):ti,ab,kw

#18 (naive bayes):ti,ab,kw

#19 (bayesian learning):ti,ab,kw

#20 (neural network*):ti,ab,kw

#21 (natural language processing):ti,ab,kw

#22 (support vector*):ti,ab,kw

#23 (random forest*):ti,ab,kw

#24 (boosting):ti,ab,kw

For peer review only

#25 (deep learning):ti,ab,kw

#26 (machine intelligence):ti,ab,kw

#27 (computational intelligence):ti,ab,kw

#28 (computer reasoning):ti,ab,kw

#29 #1 or #2 or #3 or #4 or #5 or #6 or #7 or #8 or #9 or #10 or #11 or #12 or #13 or #14 or #15 or #16 or #17 or #18 or #19 or #20 or #21 or #22 or #23 or #24 or #25 or #26 or #27 or #28

#30 MeSH descriptor: [Tomography] this term only MeSH

#31 MeSH descriptor: [Colonography, Computed Tomographic] this term only MeSH

#32 MeSH descriptor: [Computed Tomography Angiography] this term only MeSH

#33 MeSH descriptor: [Positron-Emission Tomography] this term only MeSH

#34 MeSH descriptor: [Positron Emission Tomography Computed Tomography] this term only MeSH

#35 MeSH descriptor: [Magnetic Resonance Imaging] this term only MeSH

#36 MeSH descriptor: [Cholangiopancreatography, Magnetic Resonance] this term only MeSH

#37 MeSH descriptor: [Diffusion Magnetic Resonance Imaging] this term only MeSH

#38 MeSH descriptor: [Diffusion Tensor Imaging] this term only MeSH

#39 MeSH descriptor: [Echo-Planar Imaging] this term only MeSH

#40 MeSH descriptor: [Fluorine-19 Magnetic Resonance Imaging] this term only MeSH

#41 MeSH descriptor: [Magnetic Resonance Angiography] this term only MeSH

#42 MeSH descriptor: [Magnetic Resonance Imaging, Cine] this term only MeSH

#43 MeSH descriptor: [Multiparametric Magnetic Resonance Imaging] this term only MeSH

For peer review only

#44 (MRI):ti,ab,kw

#45 (magnetic resonance imaging):ti,ab,kw

#46 #30 or #31 or #32 or #33 or #34 or #35 or #36 or #37 or #38 or #39 or #40 or #41 or #42 or #43 or #44 or #45

#47 MeSH descriptor: [Pelvis] this term only MeSH

#48 MeSH descriptor: [Lesser Pelvis] this term only MeSH

#49 MeSH descriptor: [Pelvic Floor] this term only MeSH

#50 ((radiography NEAR/3 abdominal)):ti,ab,kw

#51 ((radiography NEAR/3 abdomen)):ti,ab,kw

#52 ((Abdomen or abdominal cavity or peritoneum or douglas' pouch or mesentery or mesocolon or omentum or peritoneal cavity or peritoneal stomata or retroperitoneal space)):ti,ab,kw

#53 (pelvis or pelvic floor):ti,ab,kw

#54 #47 or #48 or #49 or #50 or #51 or #52 or #53

#55 #29 and #46 and #54

For peer review only

PRISMA-P (Preferred Reporting Items for Systematic review and Meta-Analysis Protocols) 2015 checklist: recommended items to address in a systematic review protocol*

Section and topic	Item No	Checklist item	
**ADMINISTRATIVE INFORMATION**			
Title:			
Identification	1a	Identify the report as a protocol of a systematic review	1
Update	1b	If the protocol is for an update of a previous systematic review, identify as such	
Registration	2	If registered, provide the name of the registry (such as PROSPERO) and registration number	5 & 9
Authors:			
Contact	3a	Provide name, institutional affiliation, e-mail address of all protocol authors; provide physical mailing address of corresponding author	1-2
Contributions	3b	Describe contributions of protocol authors and identify the guarantor of the review	
Amendments	4	If the protocol represents an amendment of a previously completed or published protocol, identify as such and list changes; otherwise, state plan for documenting important protocol amendments	NA
Support:			
Sources	5a	Indicate sources of financial or other support for the review	3
Sponsor	5b	Provide name for the review funder and/or sponsor	
Role of sponsor or funder	5c	Describe roles of funder(s), sponsor(s), and/or institution(s), if any, in developing the protocol	
**INTRODUCTION**			
Rationale	6	Describe the rationale for the review in the context of what is already known	7-9
Objectives	7	Provide an explicit statement of the question(s) the review will address with reference to participants, interventions, comparators, and outcomes (PICO)	8-9
**METHODS**			
Eligibility criteria	8	Specify the study characteristics (such as PICO, study design, setting, time frame) and report characteristics (such as years considered, language, publication status) to be used as criteria for eligibility for the review	10-11
Information sources	9	Describe all intended information sources (such as electronic databases, contact with study authors, trial registers or other grey literature sources) with planned dates of coverage	11-12
Search strategy	10	Present draft of search strategy to be used for at least one electronic database, including planned limits, such that it could be repeated	12
Study records:			
Data management	11a	Describe the mechanism(s) that will be used to manage records and data throughout the review	12-13

For peer review only

Selection process	11b	State the process that will be used for selecting studies (such as two independent reviewers) through each phase of the review (that is, screening, eligibility and inclusion in meta-analysis)	12-13
Data collection process	11c	Describe planned method of extracting data from reports (such as piloting forms, done independently, in duplicate), any processes for obtaining and confirming data from investigators	
Data items	12	List and define all variables for which data will be sought (such as PICO items, funding sources), any pre-planned data assumptions and simplifications	
Outcomes and prioritization	13	List and define all outcomes for which data will be sought, including prioritization of main and additional outcomes, with rationale	11
Risk of bias in individual studies	14	Describe anticipated methods for assessing risk of bias of individual studies, including whether this will be done at the outcome or study level, or both; state how this information will be used in data synthesis	13
Data synthesis	15a	Describe criteria under which study data will be quantitatively synthesised	
	15b	If data are appropriate for quantitative synthesis, describe planned summary measures, methods of handling data and methods of combining data from studies, including any planned exploration of consistency (such as I^2, Kendall's τ)	13
	15c	Describe any proposed additional analyses (such as sensitivity or subgroup analyses, meta-regression)	
	15d	If quantitative synthesis is not appropriate, describe the type of summary planned	
Meta-bias(es)	16	Specify any planned assessment of meta-bias(es) (such as publication bias across studies, selective reporting within studies)	13
Confidence in cumulative evidence	17	Describe how the strength of the body of evidence will be assessed (such as GRADE)	13

*** It is strongly recommended that this checklist be read in conjunction with the PRISMA-P Explanation and Elaboration (cite when available) for important clarification on the items. Amendments to a review protocol should be tracked and dated. The copyright for PRISMA-P (including checklist) is held by the PRISMA-P Group and is distributed under a Creative Commons Attribution Licence 4.0.**

From: Shamseer L, Moher D, Clarke M, Ghersi D, Liberati A, Petticrew M, Shekelle P, Stewart L, PRISMA-P Group. Preferred reporting items for systematic review and meta-analysis protocols (PRISMA-P) 2015: elaboration and explanation. BMJ. 2015 Jan 2;349(jan02 1):g7647.

For peer review only

BMJ Open

Artificial intelligence as a diagnostic aid in cross-sectional radiological imaging of the abdominopelvic cavity: A protocol for a systematic review

Journal:	*BMJ Open*
Manuscript ID	bmjopen-2021-054411.R1
Article Type:	Protocol
Date Submitted by the Author:	24-Aug-2021
Complete List of Authors:	Fowler, George; University of Bristol, Centre for Surgical Research, Population Health Sciences, Bristol Medical School<br>Macefield, Rhiannon; University of Bristol, Centre for Surgical Research, Population Health Sciences, Bristol Medical School<br>Hardacre, Conor; University of Bristol, Centre for Surgical Research, Population Health Sciences, Bristol Medical School<br>Callaway, Mark; Bristol Royal Infirmary, Department of Clinical Radiology<br>Smart , Neil ; Royal Devon and Exeter NHS Foundation Trust, Exeter Surgical Health Services Research Unit (HeSRU)<br>Blencowe, Natalie; University of Bristol, Centre for Surgical Research, Population Health Sciences, Bristol Medical School
**Primary Subject Heading**:	Surgery
Secondary Subject Heading:	Radiology and imaging, Diagnostics
Keywords:	Diagnostic radiology < RADIOLOGY & IMAGING, SURGERY, Computed tomography < RADIOLOGY & IMAGING, Magnetic resonance imaging < RADIOLOGY & IMAGING

SCHOLARONE™
Manuscripts

I, the Submitting Author has the right to grant and does grant on behalf of all authors of the Work (as defined in the below author licence), an exclusive licence and/or a non-exclusive licence for contributions from authors who are: i) UK Crown employees; ii) where BMJ has agreed a CC-BY licence shall apply, and/or iii) in accordance with the terms applicable for US Federal Government officers or employees acting as part of their official duties; on a worldwide, perpetual, irrevocable, royalty-free basis to BMJ Publishing Group Ltd ("BMJ") its licensees and where the relevant Journal is co-owned by BMJ to the co-owners of the Journal, to publish the Work in this journal and any other BMJ products and to exploit all rights, as set out in our licence.

The Submitting Author accepts and understands that any supply made under these terms is made by BMJ to the Submitting Author unless you are acting as an employee on behalf of your employer or a postgraduate student of an affiliated institution which is paying any applicable article publishing charge ("APC") for Open Access articles. Where the Submitting Author wishes to make the Work available on an Open Access basis (and intends to pay the relevant APC), the terms of reuse of such Open Access shall be governed by a Creative Commons licence – details of these licences and which Creative Commons licence will apply to this Work are set out in our licence referred to above.

Other than as permitted in any relevant BMJ Author's Self Archiving Policies, I confirm this Work has not been accepted for publication elsewhere, is not being considered for publication elsewhere and does not duplicate material already published. I confirm all authors consent to publication of this Work and authorise the granting of this licence.

For peer review only

Artificial intelligence as a diagnostic aid in cross-sectional radiological imaging of the abdominopelvic cavity: A protocol for a systematic review

Authors: George E Fowler[1]

George.fowler@bristol.ac.uk

Rhiannon C Macefield[1]

r.macefield@bristol.ac.uk

Conor Hardacre[1]

ch17008@bristol.ac.uk

Mark P Callaway[2]

Mark.Callaway@uhbw.nhs.uk

Neil J Smart[3]

n.smart@nhs.net

Natalie S Blencowe[1]

natalie.blencowe@bristol.ac.uk

Corresponding author: George E Fowler

University of Bristol, Centre for Surgical Research, Population Health Sciences, Bristol Medical School.

For peer review only

Institutions: [1]University of Bristol, Centre for Surgical Research, Population Health Sciences, Bristol Medical School.

[2]Department of Clinical Radiology, Bristol Royal Infirmary, Bristol, UK.

[3]Exeter Surgical Health Services Research Unit (HeSRU), Royal Devon and Exeter Hospital, Exeter, UK.

ORCID ID(s):

GEF (0000-0002-4133-802X)

RCM (0000-0002-6606-5427)

CH (0000-0001-9800-0479)

MPC

NJS (0000-0002-3043-8324)

NSB (0000-0002-6111-2175)

Author contributions: GEF and NSB conceived the idea for this systematic review. The search strategy was developed by all authors. GEF drafted the manuscript protocol (guarantor of review), and it was critically appraised and revised by RCM, CH, MPC, NJS and NSB. All authors approved the final manuscript before submission.

Acknowledgements: The authors would like to thank Catherine Borwick, our professional librarian, for her expertise in developing the search strategy. The authors are also grateful to Dr

For peer review only

Christin Hoffmann for leading patient and public feedback sessions as part of the wider programme of this research.

Funding statement: This work was supported by the Medical Research Council (MR/S001751/1).

Conflict of Interest: The authors have no conflicts of interest to declare.

Word count: 1,351 words

For peer review only

ABSTRACT

Introduction: The application of artificial intelligence (AI) technologies as a diagnostic aid in healthcare is increasing. Benefits include applications to improve health systems, such as rapid and accurate interpretation of medical images. This may improve the performance of diagnostic, prognostic and management decisions. While a large amount of work has been undertaken discussing the role of AI little is understood regarding the performance of such applications in the clinical setting. This systematic review aims to critically appraise the diagnostic performance of AI algorithms to identify disease from cross-sectional radiological images of the abdominopelvic cavity, to identify current limitations and inform future research.

Methods and analysis: A systematic search will be conducted on Medline, EMBASE and the Cochrane Central Register of Controlled Trials (CENTRAL) to identify relevant studies. Primary studies where AI-based technologies have been used as a diagnostic aid in cross-sectional radiological images of the abdominopelvic cavity will be included. Diagnostic accuracy of AI models, including reported sensitivity, specificity, predictive values, likelihood ratios and the area under the receiver operating characteristic curve will be examined and compared to standard practice. Risk of bias of included studies will be assessed using the QUADAS-2 tool. Findings will be reported according to the Synthesis Without Meta-analysis (SWiM) guidelines.

Ethics and dissemination: No ethical approval is required as primary data will not be collected. The results will inform further research studies in this field. Findings will be

For peer review only

disseminated at relevant conferences, on social media and published in a peer-reviewed journal.

PROSPERO registration number: CRD42021237249.

Key words: Systematic review, artificial intelligence, diagnostic accuracy, radiological imaging, abdominopelvic cavity.

For peer review only

BOX: Strengths and limitations of this study

- This will be the first systematic review to evaluate the diagnostic performance of AI models using cross-sectional radiological images of the abdominopelvic cavity, identifying current limitations and evidence gaps, and thereby focusing future research efforts.
- Robust methodology will be undertaken including duplicate screening, data extraction and of risk of bias assessment.
- Findings may be limited by the inclusion of English language publications only.

For peer review only

INTRODUCTION

In an era of 'Big Data', rapid developments in artificial intelligence (AI)-based technologies in medicine offer great potential to transform healthcare and improve patient outcomes [1]. The widespread adoption of digital data in healthcare has provided a vast amount of data to enable computer algorithms to extract relevant information and recognise complex patterns [2]. This includes quantitative (e.g. laboratory values) and qualitative (e.g. text-based electronic health records) data, as well as audio-visual data obtained from recordings from medical devices (e.g. electrocardiograms, digital dictation, etc). In a recent review, AI technologies were summarised as having an impact at three levels; clinicians, health systems and patients [3]. For clinicians, AI technologies can help interpret images more rapidly and accurately improving the performance of diagnostic, prognostic and management decisions [4]. For health systems, AI applications can improve workflow (e.g. administrative jobs such as scheduling of operating rooms and clinic appointments). For patients, AI technologies can provide an opportunity for individuals to process their own data to promote health (e.g. a smartwatch algorithm to detect a heart arrhythmia and the patient seeking appropriate medical attention) [3].

Medical imaging is considered a valuable source of diagnostic, prognostic and surveillance information. It also provides a pivotal role in supporting clinicians to perform procedural tasks. Images, however, have traditionally been dependent on human interpretation and there is an increasingly limited number of interpreters [5]. There has been a surge of research exploring how AI technologies can be applied to medical images to support clinicians and provide greater efficacy and efficiency in

For peer review only

clinical care [6]. One of the most promising clinical applications of AI has been in diagnostic imaging [7], particularly for radiological [7–14] and endoscopic [15,16] investigations. AI diagnostic models have been used to detect pulmonary nodules [12], liver lesions [8], pancreatic cancer [9], colorectal cancer [15] and hip fractures [13,14]. These advancements can be stratified by imaging modality (e.g., ultrasound, radiography, computed tomography (CT), magnetic resonance imaging (MRI)) and anatomical region (e.g. head and neck, thorax, abdomen and pelvis, upper and lower limbs) [17], organ [7] or speciality. However, most AI studies are currently a proof-of-concept, rather than a model deployed in the clinical setting to explore the potential benefit. Few prospective studies and RCTs evaluating the application of AI have been undertaken, and those which exist are at high risk of bias [18].

Evidence from some clinical specialities, including neurosurgery [19] and gastroenterology [20] offer insight into some of the promises and pitfalls for AI technologies in healthcare. Pitfalls include AI algorithms that can be difficult or impossible to interpret (referred to as "black box" techniques) and requiring large amounts of high quality data which can be difficult to access, especially across institutions [19]. A review of the diagnostic accuracy of AI in radiological imaging of the abdominopelvic cavity is lacking. This could benefit a variety of different surgical specialities which employ diagnostic imaging for the abdominopelvic cavity. This systematic review aims to summarise the current research and critically appraise the diagnostic performance of AI models to diagnose disease from cross-sectional radiological images of the abdominopelvic cavity which may warrant an 'invasive procedure' [21] for 'therapeutic intent'. This will be compared to standard practice. The

For peer review only

quality of this research will also be assessed, to identify current limitations and inform future research efforts.

For peer review only

METHODS

Protocol and registration

This protocol has been developed in accordance with the Preferred Reporting Items for Systematic Reviews and Meta-analysis Protocols (PRISMA-P; 'supplementary file') guidelines [22]. The systematic review has been registered with the International Prospective Register of Systematic Reviews (PROSPERO; CRD42021237249).

Eligibility criteria

Primary research studies will be considered for eligibility using the PIRT framework (participants, index test(s), reference standard and target condition) [23]:

Participants

Adults with abdominopelvic cavity pathology diagnosed from cross-sectional radiological imaging confined to CT, MRI and positron emission tomography (PET). Studies reporting endoscopy as an imaging modality will not be included in this review, as several reviews have already explored the performance of AI in this area [16,24].

Index test

Studies considering AI models as an intervention with the aim to provide a diagnosis.

Reference standard

Standard practice.

Target condition

For peer review only

Abdominopelvic cavity pathology which has had, or may warrant, an 'invasive procedure' [21] for 'therapeutic intent'.

Exclusion criteria for the studies as follows:

1. Secondary research studies (e.g. editorials and systematic reviews), case reports and case series.
2. Absence of full text publications (e.g. conference abstracts).
3. Non-English articles.
4. Animal studies.

Outcome

The primary outcome is to evaluate the diagnostic performance of AI models using cross-sectional radiological images of the abdominopelvic cavity. The diagnostic performance will be referred to as previously defined: "the ability of a test to discriminate between the target condition and health" [25]. Diagnostic measures of accuracy will include reported sensitivity, specificity and the area under the receiver operating characteristic curve (AUC).

Information sources

An electronic search of OVID SP versions of Medline, EMBASE and the Cochrane Central Register of Controlled Trials (CENTRAL) will identify all potentially relevant studies published since January 1st, 2012, using a pre-defined search strategy (Appendix S1). The cut-off from January 1st, 2012, is to accommodate for the advancement in machine learning performance with the development of deep learning approaches, an approach previously adopted in the literature [5].

For peer review only

Search strategy and study selection

The search syntax will be developed with guidance from a professional librarian using free text and Medical Subject Headings (MeSH) related to “artificial intelligence”, “diagnostic imaging” and the “abdominopelvic cavity” (Appendix S1). Database search results will be imported into the EndNote reference management software and duplicates will be removed.

Assessment of study eligibility will be a two-stage process. First, titles and abstracts will be screened for inclusion by two independent reviewers. Any identified conflicts will be resolved through discussion, including with the wider study team if required. Final eligibility will be assessed by full-text review of potentially eligible studies by the same process. The screening will be facilitated by Rayyan software [26]. Reference lists of included studies will also be assessed for study eligibility.

Data extraction and management

Eligible studies will undergo data extraction by two independent reviewers using a pre-designed standardized proforma and data management software (REDCap version 9.5.23). A standardized form will be used, which will include the following categories:

1. Study characteristics: first author, journal, year of publication, country of origin, study design (e.g. case control, randomised control trials etc) reporting of ethical approval, regulatory approval (e.g. Medicines and Healthcare products Regulatory Agency) and patient and public involvement.
2. Patient characteristics: pathology studied and surgical speciality of pathology.

For peer review only

3. Input features: modality of radiological imaging (e.g. CT, MRI and PET), AI model used, size of training model, comparator group used and size of this data set.
4. Outcomes: diagnostic measures of accuracy and method of validation.

Risk of bias

Risk of bias of the primary diagnostic accuracy studies will be assessed using the QUADAS-2 tool [27]. This will be done independently by at least two authors of the study and disagreements resolved by the study team.

Data Synthesis

Search results and study selection will be presented in accordance with the Preferred Reporting Items for Systematic Reviews (PRISMA) guidelines [28]. Due to the broad nature of the PIRT and anticipated high levels of heterogeneity for the primary outcome a meta-analysis of data is not planned. Findings will be presented as a descriptive summary and narrative synthesis and will be reported according to the Synthesis Without Meta-analysis guidelines [29]. The narrative synthesis will focus on the primary outcome with studies grouped by the modality of cross-sectional radiological imaging, pathology studied and surgical subspecialty.

Patient and public involvement

As part of the wider programme of work (Bristol Biomedical Research Centre, NIHR Bristol BRC), patients and the public were consulted on their views of AI being used to guide doctors to make decisions about treatment. Patient and public involvement (PPI) will be sought for the dissemination of this systematic review.

For peer review only

Ethics and dissemination

Ethical approval is not required for the systematic review, as no primary data is collected. The review will be disseminated at relevant conferences, on social media and published in a peer-review journal.

For peer review only

References

1 He J, Baxter SL, Xu J, *et al.* The practical implementation of artificial intelligence technologies in medicine. *Nat Med* 2019;**25**:30–6. doi:10.1038/s41591-018-0307-0

2 Alloghani M, Al-Jumeily D, Aljaaf AJ, *et al.* The Application of Artificial Intelligence Technology in Healthcare: A Systematic Review. *Commun Comput Inf Sci* 2020;**1174 CCIS**:248–61. doi:10.1007/978-3-030-38752-5_20

3 Topol EJ. High-performance medicine: the convergence of human and artificial intelligence. *Nat Med* 2019;**25**:44–56. doi:10.1038/s41591-018-0300-7

4 Ben-Israel D, Jacobs WB, Casha S, *et al.* The impact of machine learning on patient care: A systematic review. *Artif Intell Med* 2020;**103**:101785. doi:10.1016/j.artmed.2019.101785

5 Liu X, Faes L, Kale AU, *et al.* A comparison of deep learning performance against health-care professionals in detecting diseases from medical imaging: a systematic review and meta-analysis. *Lancet Digit Heal* 2019;**1**:e271–97. doi:10.1016/S2589-7500(19)30123-2

6 Hosny A, Parmar C, Quackenbush J, *et al.* Artificial intelligence in radiology. *Nat Rev Cancer* 2018;**18**:500–10. doi:10.1038/s41568-018-0016-5.Artificial

7 Lundervold AS, Lundervold A. An overview of deep learning in medical imaging focusing on MRI. *Z Med Phys* 2019;**29**:102–27. doi:10.1016/j.zemedi.2018.11.002

8 Azer SA. Deep learning with convolutional neural networks for identification of liver masses and hepatocellular carcinoma: A systematic review. *World J Gastrointest Oncol* 2019;**11**:1218–30. doi:10.4251/wjgo.v11.i12.1218

9 Liu KL, Wu T, Chen PT, *et al.* Deep learning to distinguish pancreatic cancer

For peer review only

tissue from non-cancerous pancreatic tissue: a retrospective study with cross-racial external validation. *Lancet Digit Heal* 2020;**2**:e303–13. doi:10.1016/S2589-7500(20)30078-9

10 Lakshmanaprabu SK, Mohanty SN, Shankar K, *et al.* Optimal deep learning model for classification of lung cancer on CT images. *Futur Gener Comput Syst* 2019;**92**:374–82. doi:10.1016/j.future.2018.10.009

11 Titano JJ, Badgeley M, Schefflein J, *et al.* Automated deep-neural-network surveillance of cranial images for acute neurologic events. *Nat Med* 2018;**24**:1337–41. doi:10.1038/s41591-018-0147-y

12 Nam JG, Park S, Hwang EJ, *et al.* Development and validation of deep learning-based automatic detection algorithm for malignant pulmonary nodules on chest radiographs. *Radiology* 2019;**290**:218–28. doi:10.1148/radiol.2018180237

13 Gale W, Oakden-Rayner L, Carneiro G, *et al.* Detecting hip fractures with radiologist-level performance using deep neural networks. Published Online First: 2017.http://arxiv.org/abs/1711.06504

14 Lindsey R, Daluiski A, Chopra S, *et al.* Deep neural network improves fracture detection by clinicians. *Proc Natl Acad Sci U S A* 2018;**115**:11591–6. doi:10.1073/pnas.1806905115

15 Zhou D, Tian F, Tian X, *et al.* Diagnostic evaluation of a deep learning model for optical diagnosis of colorectal cancer. *Nat Commun* 2020;**11**:1–9. doi:10.1038/s41467-020-16777-6

16 Hassan C, Spadaccini M, Iannone A, *et al.* Performance of artificial intelligence for colonoscopy regarding adenoma and polyp detection: a meta-analysis. *Gastrointest Endosc* Published Online First: 2020.

For peer review only

doi:10.1016/j.gie.2020.06.059

17 Pesapane F, Codari M, Sardanelli F. Artificial intelligence in medical imaging: threat or opportunity? Radiologists again at the forefront of innovation in medicine. *Eur Radiol Exp* 2018;**2**. doi:10.1186/s41747-018-0061-6

18 Raffort J, Adam C, Carrier M, *et al.* Artificial intelligence in abdominal aortic aneurysm. *J Vasc Surg* 2020;**72**:321-333.e1. doi:10.1016/j.jvs.2019.12.026

19 Senders JT, Arnaout O, Karhade A V., *et al.* Natural and artificial intelligence in neurosurgery: A systematic review. *Clin Neurosurg* 2018;**83**:181–92. doi:10.1093/neuros/nyx384

20 Yang YJ, Bang CS. Application of artificial intelligence in gastroenterology. *World J Gastroenterol* 2019;**25**:1666–83. doi:10.3748/wjg.v25.i14.1666

21 Cousins S, Blencowe NS, Blazeby JM. What is an invasive procedure? A definition to inform study design, evidence synthesis and research tracking. *BMJ Open* 2019;**9**:2018–20. doi:10.1136/bmjopen-2018-028576

22 Moher D, Shamseer L, Clarke M, *et al.* Preferred reporting items for systematic review and meta-analysis protocols (PRISMA-P) 2015 statement. *Syst Rev* 2015;**4**:1–9.

23 Jj, Deeks, S, Wisniewski DC, Diagnostic C, Accuracy T, *et al.* Guide to the contents of a Cochrane Diagnostic Test Accuracy Protocol. 2013;:1–15.

24 Lui TKL, Tsui VWM, Leung WK. Accuracy of artificial intelligence–assisted detection of upper GI lesions: a systematic review and meta-analysis. *Gastrointest Endosc* 2020;**92**:821-830.e9. doi:10.1016/j.gie.2020.06.034

25 Simundic AM. Measures of Diagnostic Accuracy: Basic Definitions. *EJIFCC* 2009;**19**:203–11.https://www.ncbi.nlm.nih.gov/pubmed/27683318%0Ahttps://www.ncbi.nlm.n

For peer review only

ih.gov/pmc/articles/PMC4975285/pdf/ejifcc-19-203.pdf

26 Ouzzani M, Hammady H, Fedorowicz Z, *et al.* Rayyan-a web and mobile app for systematic reviews. *Syst Rev* 2016;**5**:1–10. doi:10.1186/s13643-016-0384-4

27 Whiting PF, Rutjes AW, Westwood ME, Mallett S, Deeks JJ, Reitsma JB, Leeflang MM, Sterne JA BP. QUADAS-2 Group. QUADAS-2: a revised tool for the quality assessment of diagnostic accuracy studies. *Ann Intern Med* 2011;**8**:29–36. doi:0.7326/0003-4819-155-8-201110180-00009. PMID: 22007046.

28 McInnes MDF, Moher D, Thombs BD, *et al.* Preferred Reporting Items for a Systematic Review and Meta-analysis of Diagnostic Test Accuracy Studies The PRISMA-DTA Statement. *JAMA - J Am Med Assoc* 2018;**319**:388–96. doi:10.1001/jama.2017.19163

29 Campbell M, McKenzie JE, Sowden A, *et al.* Synthesis without meta-analysis (SWiM) in systematic reviews: Reporting guideline. *BMJ* 2020;**368**:1–6. doi:10.1136/bmj.l6890

For peer review only

APPENDIX S1

Artificial intelligence as a diagnostic aid in cross-sectional imaging of the abdominopelvic cavity: A protocol for a systematic review

Medline Search

1. artificial intelligence/ or machine learning/ or deep learning/ or supervised machine learning/ or support vector machine/ or unsupervised machine learning/
2. computer heuristics/ or expert systems/ or fuzzy logic/ or knowledge bases/ or biological ontologies/ or gene ontology/ or natural language processing/ or neural networks, computer/ or robotics/
3. Machine learning.ti,ab,kf.
4. Artificial Intelligence.ti,ab,kf.
5. Naive Bayes.ti,ab,kf.
6. bayesian learning.ti,ab,kf.
7. Neural network*.ti,ab,kf.
8. Natural language processing.ti,ab,kf.
9. support vector*.ti,ab,kf.
10. random forest*.ti,ab,kf.
11. boosting.ti,ab,kf.
12. deep learning.ti,ab,kf.
13. machine intelligence.ti,ab,kf.
14. computational intelligence.ti,ab,kf.
15. computer reasoning.ti,ab,kf.
16. 1 or 2 or 3 or 4 or 5 or 6 or 7 or 8 or 9 or 10 or 11 or 12 or 13 or 14 or 15

For peer review only

17. tomography/ or colonography, computed tomographic/ or computed tomography angiography/ or positron emission tomography computed tomography/

18. magnetic resonance imaging/ or cholangiopancreatography, magnetic resonance/ or diffusion magnetic resonance imaging/ or diffusion tensor imaging/ or echo-planar imaging/ or fluorine-19 magnetic resonance imaging/ or magnetic resonance angiography/ or magnetic resonance imaging, cine/ or multiparametric magnetic resonance imaging/

19. (MRI or Magnetic Resonance Imaging).ti,ab,kf.

20. 17 or 18 or 19

21. pelvis/ or lesser pelvis/ or pelvic floor/

22. (radiography adj3 abdominal).ti,ab,kf.

23. (Abdomen or abdominal cavity or peritoneum or douglas' pouch or mesentery or mesocolon or omentum or peritoneal cavity or peritoneal stomata or retroperitoneal space).ti,ab,kf.

24. (Pelvis or pelvic floor).ti,ab,kf.

25. 21 or 22 or 23 or 24

26. exp Animals/ not Humans/

27. (animal model* or rat or rats or mouse or mice or rodent* or sheep or lambs or murine or pigs or piglets or swine or porcine or rabbit or rabbits or cat or cats or feline or dog or dogs or canine or cattle or bovine or marmoset* or monkey or monkeys or trout or zebra fish*).ti.

28. (Comment or editorial or letter or case reports).pt.

29. (endoscope or gastroscope or colonoscope or capsule endoscopy or endoscopy).ti,ab,kf.

30. 26 or 27 or 28 or 29

For peer review only

31. 16 and 20 and 25

32. 31 not 30

33. (2012* or 2013* or 2014* or 2015* or 2016* or 2017* or 2018* or 2019* or 2020* or 202101*).yr,ed,dc,ep.

34. 32 and 33

For peer review only

Embase via Ovid

1. artificial intelligence/ or ambient intelligence/ or automated reasoning/ or computer heuristics/ or multicriteria decision analysis/

2. exp machine learning/

3. deep learning/

4. supervised machine learning/

5. exp support vector machine/

6. unsupervised machine learning/

7. hyperheuristics/ or metaheuristics/

8. expert systems/ or fuzzy logic/ or knowledge bases/ or natural language processing/ or neural networks, computer/ or robotics/

9. biological ontologies/ or gene ontology/

10. ((machine or bayesian or deep) adj1 learning).ti,ab,kw.

11. ((artificial or machine or computational) adj1 intelligence).ti,ab,kw.

12. Naive Bayes.ti,ab,kw.

13. Neural network*.ti,ab,kw.

14. Natural language processing.ti,ab,kw.

15. support vector*.ti,ab,kw.

16. random forest*.ti,ab,kw.

17. boosting.ti,ab,kw.

18. computer reasoning.ti,ab,kw.

19. 1 or 2 or 3 or 4 or 5 or 6 or 7 or 8 or 9 or 10 or 11 or 12 or 13 or 14 or 15 or 16 or 17 or 18

20. exp Tomography/

For peer review only

21. colonography, computed tomographic/ or computed tomography angiography/ or four-dimensional computed tomography/ or positron emission tomography computed tomography/ or single photon emission computed tomography computed tomography/ or tomography, spiral computed/

22. magnetic resonance imaging/ or cholangiopancreatography, magnetic resonance/ or diffusion magnetic resonance imaging/ or diffusion tensor imaging/ or echo-planar imaging/ or fluorine-19 magnetic resonance imaging/ or magnetic resonance angiography/ or magnetic resonance imaging, cine/ or multiparametric magnetic resonance imaging/

23. (MRI or Magnetic Resonance Imaging).ti,ab,kw.

24. 20 or 21 or 22 or 23

25. pelvis/ or lesser pelvis/ or pelvic floor/

26. (radiography adj3 abdominal).ti,ab,kw.

27. (Abdomen or abdominal cavity or peritoneum or douglas' pouch or mesentery or mesocolon or omentum or peritoneal cavity or peritoneal stomata or retroperitoneal space).ti,ab,kw.

28. (Pelvis or pelvic floor).ti,ab,kw.

29. 25 or 26 or 27 or 28

30. (Animal/ or Nonhuman/) not Human/

31. Animal Experiment/ not (Human Experiment/ or Human/)

32. (animal model* or rat or rats or mouse or mice or rodent* or sheep or lambs or murine or pigs or piglets or swine or porcine or rabbit or rabbits or cat or cats or feline or dog or dogs or canine or cattle or bovine or marmoset* or monkey or monkeys or trout or zebra fish*).ti.

33. (Comment or editorial or letter or case reports).pt.

For peer review only

34. (endoscope or gastroscope or colonoscope or capsule endoscopy or endoscopy).ti,ab,kw.

35. 30 or 31 or 32 or 33 or 34

36. 19 and 24 and 29

37. 36 not 35

38. (2012* or 2013* or 2014* or 2015* or 2016* or 2017* or 2018* or 2019* or 2020* or 202101*).yr,em.

39. 37 and 38

For peer review only

Cochrane Central Register of Controlled Trials

#1 MeSH descriptor: [Artificial Intelligence] this term only MeSH

#2 MeSH descriptor: [Machine Learning] this term only MeSH

#3 MeSH descriptor: [Deep Learning] this term only MeSH

#4 MeSH descriptor: [Supervised Machine Learning] this term only MeSH

#5 MeSH descriptor: [Unsupervised Machine Learning] this term only MeSH

#6 MeSH descriptor: [Support Vector Machine] this term only MeSH

#7 MeSH descriptor: [Computer Heuristics] this term only MeSH

#8 MeSH descriptor: [Expert Systems] this term only MeSH

#9 MeSH descriptor: [Fuzzy Logic] this term only MeSH

#10 MeSH descriptor: [Knowledge Bases] this term only MeSH

#11 MeSH descriptor: [Biological Ontologies] this term only MeSH

#12 MeSH descriptor: [Gene Ontology] this term only MeSH

#13 MeSH descriptor: [Natural Language Processing] this term only MeSH

#14 MeSH descriptor: [Neural Networks, Computer] this term only MeSH

#15 MeSH descriptor: [Robotics] this term only MeSH

#16 (machine learning): ti,ab,kw

#17 (artificial intelligence):ti,ab,kw

#18 (naive bayes):ti,ab,kw

#19 (bayesian learning):ti,ab,kw

#20 (neural network*):ti,ab,kw

#21 (natural language processing):ti,ab,kw

#22 (support vector*):ti,ab,kw

#23 (random forest*):ti,ab,kw

#24 (boosting):ti,ab,kw

For peer review only

#25 (deep learning):ti,ab,kw

#26 (machine intelligence):ti,ab,kw

#27 (computational intelligence):ti,ab,kw

#28 (computer reasoning):ti,ab,kw

#29 #1 or #2 or #3 or #4 or #5 or #6 or #7 or #8 or #9 or #10 or #11 or #12 or #13 or #14 or #15 or #16 or #17 or #18 or #19 or #20 or #21 or #22 or #23 or #24 or #25 or #26 or #27 or #28

#30 MeSH descriptor: [Tomography] this term only MeSH

#31 MeSH descriptor: [Colonography, Computed Tomographic] this term only MeSH

#32 MeSH descriptor: [Computed Tomography Angiography] this term only MeSH

#33 MeSH descriptor: [Positron-Emission Tomography] this term only MeSH

#34 MeSH descriptor: [Positron Emission Tomography Computed Tomography] this term only MeSH

#35 MeSH descriptor: [Magnetic Resonance Imaging] this term only MeSH

#36 MeSH descriptor: [Cholangiopancreatography, Magnetic Resonance] this term only MeSH

#37 MeSH descriptor: [Diffusion Magnetic Resonance Imaging] this term only MeSH

#38 MeSH descriptor: [Diffusion Tensor Imaging] this term only MeSH

#39 MeSH descriptor: [Echo-Planar Imaging] this term only MeSH

#40 MeSH descriptor: [Fluorine-19 Magnetic Resonance Imaging] this term only MeSH

#41 MeSH descriptor: [Magnetic Resonance Angiography] this term only MeSH

#42 MeSH descriptor: [Magnetic Resonance Imaging, Cine] this term only MeSH

#43 MeSH descriptor: [Multiparametric Magnetic Resonance Imaging] this term only MeSH

For peer review only

#44 (MRI):ti,ab,kw

#45 (magnetic resonance imaging):ti,ab,kw

#46 #30 or #31 or #32 or #33 or #34 or #35 or #36 or #37 or #38 or #39 or #40 or #41 or #42 or #43 or #44 or #45

#47 MeSH descriptor: [Pelvis] this term only MeSH

#48 MeSH descriptor: [Lesser Pelvis] this term only MeSH

#49 MeSH descriptor: [Pelvic Floor] this term only MeSH

#50 ((radiography NEAR/3 abdominal)):ti,ab,kw

#51 ((radiography NEAR/3 abdomen)):ti,ab,kw

#52 ((Abdomen or abdominal cavity or peritoneum or douglas' pouch or mesentery or mesocolon or omentum or peritoneal cavity or peritoneal stomata or retroperitoneal space)):ti,ab,kw

#53 (pelvis or pelvic floor):ti,ab,kw

#54 #47 or #48 or #49 or #50 or #51 or #52 or #53

#55 #29 and #46 and #54

For peer review only

PRISMA-P (Preferred Reporting Items for Systematic review and Meta-Analysis Protocols) 2015 checklist: recommended items to address in a systematic review protocol*

Section and topic	Item No	Checklist item	
**ADMINISTRATIVE INFORMATION**			
Title:			
Identification	1a	Identify the report as a protocol of a systematic review	1
Update	1b	If the protocol is for an update of a previous systematic review, identify as such	
Registration	2	If registered, provide the name of the registry (such as PROSPERO) and registration number	5 & 9
Authors:			
Contact	3a	Provide name, institutional affiliation, e-mail address of all protocol authors; provide physical mailing address of corresponding author	1-2
Contributions	3b	Describe contributions of protocol authors and identify the guarantor of the review	
Amendments	4	If the protocol represents an amendment of a previously completed or published protocol, identify as such and list changes; otherwise, state plan for documenting important protocol amendments	NA
Support:			
Sources	5a	Indicate sources of financial or other support for the review	3
Sponsor	5b	Provide name for the review funder and/or sponsor	
Role of sponsor or funder	5c	Describe roles of funder(s), sponsor(s), and/or institution(s), if any, in developing the protocol	
**INTRODUCTION**			
Rationale	6	Describe the rationale for the review in the context of what is already known	7-9
Objectives	7	Provide an explicit statement of the question(s) the review will address with reference to participants, interventions, comparators, and outcomes (PICO)	8-9
**METHODS**			
Eligibility criteria	8	Specify the study characteristics (such as PICO, study design, setting, time frame) and report characteristics (such as years considered, language, publication status) to be used as criteria for eligibility for the review	10-11
Information sources	9	Describe all intended information sources (such as electronic databases, contact with study authors, trial registers or other grey literature sources) with planned dates of coverage	11-12
Search strategy	10	Present draft of search strategy to be used for at least one electronic database, including planned limits, such that it could be repeated	12
Study records:			
Data management	11a	Describe the mechanism(s) that will be used to manage records and data throughout the review	12-13

For peer review only

Selection process	11b	State the process that will be used for selecting studies (such as two independent reviewers) through each phase of the review (that is, screening, eligibility and inclusion in meta-analysis)	12-13
Data collection process	11c	Describe planned method of extracting data from reports (such as piloting forms, done independently, in duplicate), any processes for obtaining and confirming data from investigators	
Data items	12	List and define all variables for which data will be sought (such as PICO items, funding sources), any pre-planned data assumptions and simplifications	
Outcomes and prioritization	13	List and define all outcomes for which data will be sought, including prioritization of main and additional outcomes, with rationale	11
Risk of bias in individual studies	14	Describe anticipated methods for assessing risk of bias of individual studies, including whether this will be done at the outcome or study level, or both; state how this information will be used in data synthesis	13
Data synthesis	15a	Describe criteria under which study data will be quantitatively synthesised	
	15b	If data are appropriate for quantitative synthesis, describe planned summary measures, methods of handling data and methods of combining data from studies, including any planned exploration of consistency (such as I^2, Kendall's τ)	13
	15c	Describe any proposed additional analyses (such as sensitivity or subgroup analyses, meta-regression)	
	15d	If quantitative synthesis is not appropriate, describe the type of summary planned	
Meta-bias(es)	16	Specify any planned assessment of meta-bias(es) (such as publication bias across studies, selective reporting within studies)	13
Confidence in cumulative evidence	17	Describe how the strength of the body of evidence will be assessed (such as GRADE)	13

*** It is strongly recommended that this checklist be read in conjunction with the PRISMA-P Explanation and Elaboration (cite when available) for important clarification on the items. Amendments to a review protocol should be tracked and dated. The copyright for PRISMA-P (including checklist) is held by the PRISMA-P Group and is distributed under a Creative Commons Attribution Licence 4.0.**

From: Shamseer L, Moher D, Clarke M, Ghersi D, Liberati A, Petticrew M, Shekelle P, Stewart L, PRISMA-P Group. Preferred reporting items for systematic review and meta-analysis protocols (PRISMA-P) 2015: elaboration and explanation. BMJ. 2015 Jan 2;349(jan02 1):g7647.

For peer review only
